# Molecular Screening for Urothelial Cancer: How Close We Are?

**DOI:** 10.1055/s-0043-1768958

**Published:** 2023-05-23

**Authors:** Athanasios Michas, Basileios Michas, Anastasios Tsitsibis, Nikolaos Tsoukalas

**Affiliations:** 1 Department of Oncology, 401 General Army Hospital of Athens, 401 Geniko Stratiotiko Nosokomeio Athenon, Athina, Greece

**Keywords:** genomics, urothelial cancer, liquid biopsy

## Abstract

Early detection of urothelial cancer offers the potential for effective and successful treatment. Despite previous efforts, currently, there is not a well-validated, recommended screening program in any country. This integrative, literature-based review provides details on how recent molecular advances may further advance early tumor detection. The minimally invasive liquid biopsy is capable of identifying tumor material in human fluid samples from asymptomatic individuals. Circulating tumor biomarkers (cfDNA, exosomes, etc.) are very promising and are attracting the interest of numerous studies for the diagnosis of early-stage cancer. However, this approach definitely needs to be refined before clinical implementation. Nevertheless, despite the variety of current obstacles that require further research, the prospect of identifying urothelial carcinoma by a single urine or blood test seems truly intriguing.

## Introduction

Recent advances in molecular pathology and genomics have revolutionized research in cancer biology. The substantial increase in knowledge of tumor pathogenesis and the development of effective treatments have significantly increased survival rates. 1 2 A critical step for further reducing malignancy mortality rates is to improve early identification of primary invasive or even preinvasive lesions. Effective screening protocols have been successfully applied in breast, colorectal, and cervical cancer, and have contributed to a significant decrease in mortality rates. 3 Similarly, convincing screening methods for more malignancies would be of paramount importance and clinical value.

Population-based screening programs theoretically provide the best opportunity to reduce mortality from aggressive tumors. Effective protocols require a cost-effective, accurate test, oriented to a well-defined, specific population with a disease whose natural history and management may be modified by early detection. However, screening may also lead to over diagnosis and overtreatment of indolent disorders, cause harm to individuals and impose unnecessary costs to health care systems, or yield biased reassurance with false-negative results. Therefore, population screening is recommended only when the proven benefits outweigh the potential risks. 4

Urothelial (bladder, ureters, renal pelvis) cancer is common disease and major cause of death in the Western world. As for the majority of tumors, early detection increases chances of complete cure or prolonged survival. For instance, muscle-invasive bladder cancer and metastatic disease have disappointing outcomes compared with nonmuscle-invasive bladder cancer. 5 Therefore, implementation of diagnostic markers that could recognize tumors at primary stage is crucial. Currently, conventional cystoscopy combined with urine cytology remains the gold standard for diagnosis. Despite significant contribution in decrease of mortality, both methods suffer from several weaknesses, as screening methods. 6 On the other hand, liquid biopsy is a noninvasive and high-sensitive method capable to identify carcinomatous and precarcinomatous lesions in blood/urine/saliva of patients or asymptomatic individuals. 7 Consequently, as technological and analytical procedures evolve and cost declines, liquid biopsy holds great potential as urothelial cancer screening method. 8

## Methods

To investigate the potential role of liquid biopsy, as screening approach of urothelial carcinomas, we performed an in-depth review and analysis of the robust and valid literature. Keywords included: urothelial cancer, screening, liquid biopsy, cell-free DNA, exosomes, circulating biomarkers. To date (October 2022), ∼700 studies were found in PubMed, Medline, and the official e-library of Queen Mary College, in which 4 involved randomized controlled trials. After that, we analyzed the molecular markers and recognized the interactions between them. Finally, we concluded to an inclusive and representative gene panel.

## Discussion

Until now, screening for urothelial cancer is not recommended in any country. This is due to insufficient data to define an effective protocol oriented to an appropriate population. Despite distinguishing and stratification attempts, all previous screening approaches have failed to achieve significant clinical benefit in cost-effective way. 9 Therefore, researchers have focused on identifying individuals of high-risk groups. Vickers et al applied a decision analysis protocol based on a risk-score panel for bladder cancer (0–11). This panel incorporated well-known associated factors including age, male sex, smoking, occupational exposure, and family history. 10 Initial efforts were based on the idea of identifying microhematuria (common feature of BCA) in asymptomatic individuals with increased danger for urothelial tumors. However, outcomes from large studies evaluating hematuria dipstick examination were controversial and failed to validate an effective and convincing result. 11 Moreover, a small number of soluble urinary protein biomarkers such as nuclear matrix protein 22, bladder tumor antigen, and cell-based tests such as UroVysion (fluorescence in situ hybridization) and ImmunoCyt have been extensively studied as potential screening methods. Despite Food and Drug Administration approval, these tests are not widely used because they failed to provide health benefit in numerous clinical trials. Their use in initial identification of urothelial cancer remains limited due to low sensitivity and specificity. Currently, there are not recommendations for implementation of these markers in routine health care service. 12

Advances in molecular biology offer great opportunities to develop an effective screening program for urothelial tumors. Primarily, progress of next-generation sequencing (NGS), quantitative polymerase chain reaction (qPCR), and computational protocols allow a more comprehensive approach to elucidate bladder cancer genomics and heterogeneity in molecular level. 13 The ability to detect specific genetic markers or mutations, correlated with urothelial cancer in asymptomatic individuals through an easily applicable, cost-effective approach would be crucial step. Data from Beijing Genomic Institute and The Cancer Genome Atlas illustrate the high genetic complexity of urothelial cancers. 14

**Fig. 1:**
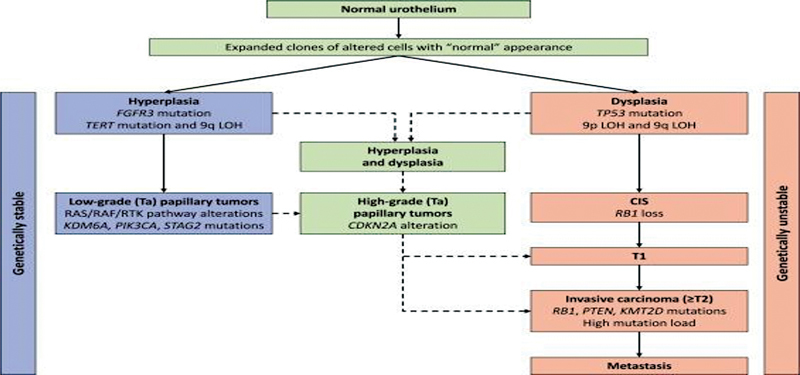
Molecular pathogenesis of urothelial cancer. 15

Urothelial tumors are characterized by frequent (hotspot) mutations in multiple molecular pathways including cell-cycle genes ( *TP53* , *CDKN2A* , *RB1* ), genes correlated with kinase signaling pathways (RTK/RAS/RAF and PI3K/AKT/mTOR) and TERT gene promoter. 15 In addition, specific gene mutations associated with epigenetic regulation, such as DNA methylation patterns, chromatin remodeling, and histone modifications seem to strongly influence urothelial tumor biology. 16 Moreover, the mutational burden seems to be significantly mediated by the APOBEC enzyme family, especially in early disease. Furthermore, alterations in untranslated regions of genome such as noncoding RNAs have been directly linked with tumor development. In addition, according to several reports, noncoding RNAs (microRNAs [miRNAs] and long noncoding RNAs) influence the expression of target genes, regulating pathways of tumorigenesis 17 18 ( Fig. 1 ).

Despite the significant advance of knowledge around urothelial cancer molecular signatures and pathways, currently, there is limited translation in screening applications. However, recently, there is growing interest toward the use of minimally invasive “liquid biopsy” to identify biomarkers in urologic malignancies. 19 Liquid biopsy refers to a single test of human fluid samples (blood, urine, saliva, etc.) capable to detect circulating tumor material. 20 This noninvasive approach may identify circulating tumor DNA (ctDNA), circulating tumor cells (CTCs), miRNAs, proteins, and exosomes. The potential utility of circulating material in urothelial cancer is further supported by the high mutational rate of this tumor. 21 Currently, most researchers focus mainly on cfDNA and exosomes, comparing to labor-intensive CTCs. The presence of ctDNA in blood was first described in mid-1970s. Despite confirmation of containing all the hallmark mutations of cancer, the low quantities in early-stage disease (<0.1–10% of total cfDNA) provided an extreme obstacle for investigation.

Nevertheless today, high-throughput sequencing and allele-specific qPCR protocols (such as multiplex and digital droplet PCR) have greatly improved the ability to detect and analyze ctDNA. 22 Theoretically, if tumor DNA is present in urine (best sample for urinary malignancies) or blood, it can be amplified by digital PCR and detect specific regions of interest. Moreover, NGS can be applied to simultaneously interrogate multiple loci of tumor genome. 23 Therefore, by implementing our current knowledge on urothelial tumors genomics to produce an efficient panel of hot-spot genes and chromosomes, representing all malignant pathways, commonly altered in urothelial cancer we could have a useful and specific tumor footprint. 24 In a primary diagnostic (not-screening) trial, researchers used a small seven-gene panel (TERT, FGFR3, TP53, PIK3CA, HRAS, KDM6A, RXRA) to detect tumor DNA in urine of 231 mixed healthy and patient individuals. 25 Overall sensitivity was 70% and specificity was 97%, with reasonable cost. Moreover, Cohen et al examined the capacity of a blood test to detect eight common cancers. The “CancerSEEK” test investigates the level of circulating proteins and mutations in cell-free DNA. It was applied in 1,005 patients with nonmetastatic tumors and performed sensitivity 69 to 98% and specificity ∼99%, depending on cancer type. 26 The authors concluded that CancerSEEK protocol definitely deserves further evaluation as potential screening approach. Furthermore, circulating exosomal DNA may be superior to cfDNA, as provides larger fragments of DNA and RNA for analysis, facilitating PCR pipeline. 27 Exosomes are tiny extracellular vesicles, generated by all living cells and contain DNA, RNA, and proteins. Studies around cancer screening suggest that exosomal DNA isolated even from minuscule serum samples is really promising but requires further validation. A combination of circulating exosomal DNA with other screening markers has potential to offer reliable sensitivity and specificity for urothelial cancer detection.

The great prospective of screening urothelial cancer with noninvasive analysis is challenging. Prior to approval, there are multiple practical, biological, and computational considerations. First, crucial step is to recognize the population group that benefits mostly from screening. So primarily, researchers focus on high-risk individuals. Apparently, liquid biopsy test should be combined with a relative phenotype and environmental factors, to create a polygenic risk score. Currently, there are risk assessment models (age, gender, smoking, exposure to amines, arsenic, etc.), but more standardization is required for the exact group that alleviates mostly. Furthermore, there are multiple technical challenges. ctDNA varies from 0.1 to 10% of cell-free DNA. Discrimination between normal and tumor DNA is extremely demanding due to the very low levels of ctDNA, even in late stages of cancer. 28 Especially for early-stage disease when detection is more likely to provide benefit, serum levels of tumor DNA may be insufficient for analysis. 29 For example, asymptomatic patients may harbor <1% mutant molecules/mL of urine, which is beyond the capacity of current NGS methods. Moreover, specificity is another crucial consideration. Mutations in genes such as *P53* can also occur in healthy individuals and provide substantial numbers of false-positive results, leading to overdiagnosis and overtreatment. 30 Therefore, multiple studies of healthy individuals are necessary to ensure accuracy. Furthermore, cost and equipment-availability issues are very important. 31 Currently, NGS and sophisticated computational methods are mostly performed in centralized laboratories, not easily accessible for screening purposes. 29 Simultaneously, despite the significant cost reduction of sequencing methods, large studies assessing the cost efficacy of circulating tumor material as screening method remain extremely important and challenging.

## Conclusion

As inference, urothelial malignancies remain one of the leading causes of cancer-related morbidity and mortality. Early detection of patients has the ability to offer extra therapeutic options and ameliorate prognosis. Currently, there is not a standardized, widely approved screening protocol, even for high-risk individuals. Despite the definite biological and technical obstacles, liquid biopsy holds great promise. The minimally invasive nature provides significant advantage for early detection. A combined use of circulating biomarkers may be ideal for urothelial cancer screening with high accuracy. Nevertheless, more studies are necessary to assess clinical utility and further standardize all analytical parameters. The future envisions exciting.
